# Acute Efficacy of Pulsed‐Field Ablation of the Mitral Isthmus Using a Pentaspline Catheter and Two Different Ablation Settings

**DOI:** 10.1111/jce.70236

**Published:** 2025-12-27

**Authors:** Josef Hornof, Marek Hozman, Dalibor Heřman, Oleksii Romanenko, Sabri Hassouna, Jana Veselá, Věra Filipcová, Lukáš Povišer, Jakub Karch, Jana Hozmanová, Lucie Znojilová, Anna Molová, Vojtěch Pěnkava, Pavel Osmančík

**Affiliations:** ^1^ Department of Cardiology Královské Vinohrady University Hospital Prague Czech Republic; ^2^ Third Faculty of Medicine Charles University Prague Czech Republic; ^3^ Cardiocenter, Karlovy Vary Hospital Karlovy Vary Czech Republic

**Keywords:** atrial fibrillation, mitral isthmus ablation, pentaspline catheter, pulsed‐field ablation, reconduction

## Abstract

**Introduction:**

There is limited data regarding the use of pulsed‐field ablation (PFA) for mitral isthmus (MI) ablation in patients with non‐paroxysmal atrial fibrillation (AF). Our aim was to assess the acute efficacy of MI ablation using a pentaspline PFA catheter with two different ablation settings.

**Methods:**

Patients with AF undergoing ablation were consecutively enrolled. All patients underwent pulmonary vein isolation and left atrial posterior wall ablation. MI ablation was performed in up to 4 series (9 PFA applications each) in Cohort A, or in up to 3 series (20 PFA applications each) in Cohort B. Each series was followed by a 20‐min observation period to verify the durability of the MI block. The primary endpoints were a first‐pass MI block and a final MI block after all ablation series.

**Results:**

Between September 2024 and June 2025, 70 patients were enrolled: 30 in Cohort A and 40 in Cohort B. A first‐pass MI block was achieved in 10 (33.3%) patients in Cohort A and 28 (70%) patients in Cohort B (*p* = 0.003). A final MI block was present in 27 (90%) and 35 (87.5%) patients in Cohort A and B, respectively (*p* = 1.00). The median reconduction time was 6 min (IQR 2.8–9.4) in Cohort A and 5 min (IQR 2.6–8.7) in Cohort B. No major complications occurred.

**Conclusion:**

Using series of 20 PFA applications increased the success rate of a first‐pass MI block to 70%. An observation period is recommended to verify the durability of MI block in PFA.

Clinical Trial Registration: NCT06803238.

**Abbreviations**
AF: arial fibrillation
AKI: acute kidney injury
CS: coronary sinus
CTI: cavotricuspid isthmus
LA: left atrium
LAPW: left atrial posterior wall
MI: mitral isthmus
PFA: pulsed‐field ablation
PVI: pulmonary veins isolation
RF: radiofrequency

## 1 Introduction

Pulmonary vein isolation (PVI) is the cornerstone of atrial fibrillation (AF) treatment. Since the efficacy of PVI is lower in patients with non‐paroxysmal AF compared to those with paroxysmal AF, additional ablation strategies are often employed for non‐paroxysmal AF. Additional linear ablation lesions, including the mitral isthmus line, have been found to improve sinus rhythm maintenance compared to PVI alone [1]. However, linear ablations are challenging because incomplete ablation lines can be pro‐arrhythmogenic and increase the risk of macro‐reentrant atrial tachycardias [2].

The mitral isthmus (MI), originally referred to as the left atrial isthmus, is a line connecting the left inferior pulmonary vein with the mitral annulus. It was first defined in 2001 by Luria et al. [3] and subsequently anatomically described by Becker and colleagues [4]. In 2004, Jaïs et al. initially demonstrated the additional benefits of mitral isthmus ablation in the context of catheter ablation for atrial fibrillation [5].

Achieving a complete block across the mitral isthmus using radiofrequency (RF) energy is challenging, and it frequently requires not only extensive endocardial ablation, but also epicardial ablation from within the coronary sinus (CS) or ethanol infusion into the vein of Marshall [6].

Pulsed‐field ablation (PFA) has recently emerged as a promising alternative to RF energy. In PVI, PFA reduces procedure time and is associated with less collateral damage, a favorable safety profile, and comparable or even better efficacy compared to other energy sources [7, 8, 9].

Despite its growing use, data regarding PFA of the MI remain limited. The objective of this reported study was to assess the acute, peri‐procedural efficacy of MI ablation using a pentaspline PFA catheter and two different ablation settings.

## 2 Methods

### 2.1 Study Design and Population

Our prospective observational study involved two consecutive cohorts of patients (Cohort A and Cohort B) with identical inclusion and exclusion criteria. Cohort A served as a pilot study, and its complete enrollment occurred before that of Cohort B. The MI ablation setting in Cohort B was based on the results of Cohort A; we report the results of both cohorts in this study.

The study (clinicaltrials.gov; NCT06803238) was conducted at two sites in the Czech Republic: a tertiary cardiac center at the University Hospital Královské Vinohrady in Prague and a regional cardiac center in Karlovy Vary. The protocols for each cohort were reviewed and approved by the local institutional ethics committees. All participants provided written informed consent before enrollment.

Consecutive patients with symptomatic non‐paroxysmal AF scheduled for catheter ablation in accordance with current guideline recommendations were prospectively enrolled. Only patients undergoing a first‐ever left atrial (LA) ablation procedure were eligible, regardless of a history of regular atrial tachycardia. As such, inclusion criteria were non‐paroxysmal AF, indication for catheter ablation, and a willingness to participate.

Exclusion criteria included left ventricular ejection fraction < 30%, a history of prior left atrial catheter ablation, and clinical conditions posing increased risk during prolonged analgosedation, such as stage III or IV chronic obstructive pulmonary disease or treated obstructive sleep apnea.

### 2.2 Procedural Workflow, Pulmonary Vein Isolation, and Left Atrial Posterior Wall Ablation

The procedural workflow was identical in both cohorts except for the MI ablation setting. The final dose of direct oral anticoagulants was given the day before the procedure: apixaban or dabigatran in the evening, and rivaroxaban in the morning. Periprocedural anticoagulation was managed with intravenous unfractionated heparin. An initial bolus of 5000 international units was administered after obtaining femoral access and before transseptal puncture, with additional boluses administered to achieve and maintain an activated clotting time of greater than 350 s. All procedures were performed under deep sedation or general anesthesia by an anesthesiologist. Vascular access was obtained under ultrasound guidance.

A steerable decapolar catheter was positioned in the CS, and transseptal access was obtained using an SL1 sheath and BRK needle (Abbott, Abbott Park, IL, USA) under combined fluoroscopic and intracardiac echocardiographic guidance. The SL1 sheath was subsequently exchanged for a steerable 16.8 F Faradrive sheath, through which a 31 mm Farawave pulsed‐field ablation catheter (Boston Scientific, Marlborough, MA, USA) was introduced.

Pulmonary vein isolation (PVI) was performed as the initial step in all patients, with eight pulsed‐field ablation (PFA) applications delivered per vein, four in the “flower” configuration and four in the “basket” configuration. Additional applications were delivered at the electrophysiologist's discretion to ensure complete PVI. Two additional anchoring applications per vein were delivered to the left atrial posterior wall (LAPW). With the guidewire left in the pulmonary vein, the ablation catheter was rotated toward the LAPW until new sharp electrograms were observed, confirming sufficient overlap with the LAPW. These anchoring lesions were counted as part of the LAPW applications. Following this, the entrance block was assessed for each of the pulmonary veins.

As the second step, the LAPW was ablated. Two rows of overlapping lesions (typically three pairs of PFA applications per row) were delivered to connect the left and right pulmonary veins. As such, the number of applications delivered to the LAPW, including the anchoring lesions, was 20. However, the final number was determined at the electrophysiologist's discretion, based on catheter stability and the anatomical length of the LAPW. All LAPW lesions were applied in the flower configuration. Durability of LAPW ablation was not assessed during the procedure. If the patient remained in AF after PVI and LAPW ablation, electrical cardioversion was performed to enable pacing maneuvers during the MI ablation.

### 2.3 Mitral Isthmus Ablation and Conduction Block Assessment

**Figure 1:**
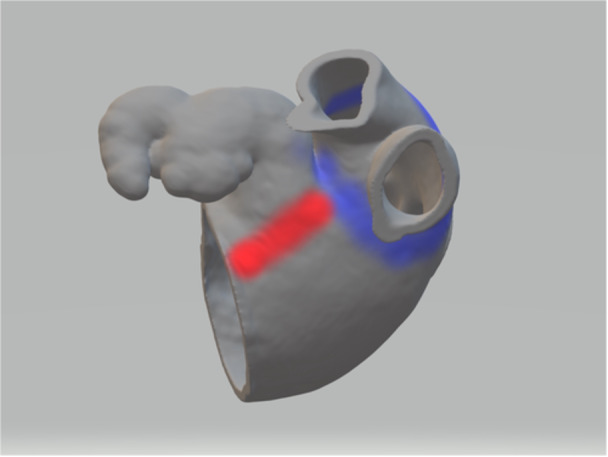
The mitral isthmus ablation line (red) connects the mitral annulus to the base of the left atrial appendage and the ablation area around the left pulmonary veins (blue).

**Figure 2:**
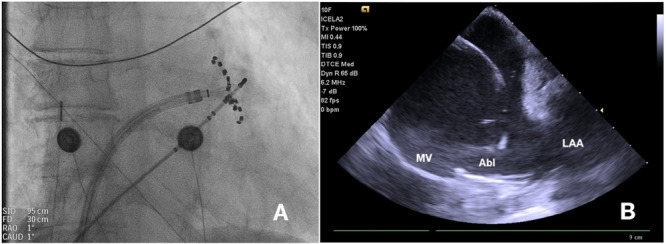
Position of the pentaspline catheter during the mitral isthmus ablation. A—Fluoroscopy antero‐posterior projection. B—Intracardiac echocardiography. Abl = ablation catheter, LAA = left atrial appendage, MV = mitral valve.

As the third step, MI ablation was conducted between the mitral annulus and the posterior base of the left atrial appendage (described in detail further) during continuous pacing from the distal CS to observe signal splitting on the pentaspline catheter. The mitral line was created slightly anterior to the conventional left posterolateral mitral isthmus line (Figures 1, 2), for two main reasons: (1) to improve the likelihood of conduction block, based on our previous unpublished experience and (2) enhanced visual control of catheter stability (i.e., maintain precise positioning) using intracardiac echocardiography. Care was taken to connect the ablation lesion not only to the base of the left atrial appendage, but also to the anterior aspect of the left pulmonary veins, ensuring completion of the MI line. The number of PFA applications and the catheter configuration differed between the two cohorts.

The goal of mitral isthmus ablation was to achieve a bidirectional conduction block, confirmed through standard differential pacing maneuvers. To accomplish this, a decapolar catheter was inserted into the CS, with the distal pole positioned posterior to the mitral ablation line. The bidirectional block was verified by (1) a concentric activation sequence in the CS during pacing from the LA appendage, and (2) an eccentric activation sequence during pacing from the distal CS pole, with the clearly latest electrical signal recorded on the pentaspline catheter positioned in the LA appendage.

### 2.4 Cohort A

At study initiation, no specific data were available regarding the optimal number of PFA applications required for an effective MI ablation. The number of applications used in Cohort A was based on early experience with posterolateral MI ablation [10].

In Cohort A, PFA was delivered using the flower configuration in sequential series of nine applications. The ablation catheter was rotated slightly (approximately 10°–25°) after every three applications to improve lesion coverage. Following ablation, the bidirectional MI block was assessed continuously for 20 min using pacing from either the distal CS or the LA appendage. If the block remained stable after 20 min, the procedure was concluded; except in patients with a history of typical atrial flutter, in whom a cavotricuspid isthmus (CTI) line was created.

If reconduction across the MI was observed, an additional series of nine PFA applications was delivered using the same protocol, followed by a repeated 20‐min observation period to reassess bidirectional block. This process was repeated up to a maximum of three times (i.e., three distinct series of nine PFA applications, totaling 27 PFA applications), each delivered using the same catheter configuration and rotation protocol. If a durable conduction block was not achieved after the third series of nine PFA applications, a fourth ablation series could be delivered at the operator's discretion. However, unlike the previous series, this final application was not protocol‐defined, that is, more than nine applications could be administered, and the catheter configuration could be changed from “flower” to “basket.” The decision to proceed with this non‐standardized lesion series was based primarily on the prior likelihood of achieving MI block, the patient's history of regular atrial tachycardia, and the anatomical characteristics of the MI. If a durable conduction block was still not achieved after the fourth ablation series, no further attempts at MI ablation were made.

Since large numbers of PFA applications can cause hemolysis and put the patient at risk of acute kidney injury (AKI) [11], and since coronary artery spasm had been described after PFA of the MI [10], the total number of PFA applications to the MI was limited to 50. For both safety and practical reasons, the total observational time was limited to 40 min. During ablation, the ST‐segment was closely monitored for possible circumflex artery spasms. According to local standards, prophylactic nitrates were not administered beforehand.

### 2.5 Cohort B

Based on the findings in Cohort A, particularly the low rate of durable first‐pass mitral isthmus block, the ablation protocol was modified and evaluated in Cohort B. The revised strategy consisted of two key changes: (1) the number of PFA applications was increased from nine to 20 per series and (2) the last four PFA applications were delivered in the basket, rather than the flower configuration.

In Cohort B, the procedure workflow mirrored that in Cohort A. Following PVI, LAPW ablation was performed. In cases of persistent AF, electrical cardioversion was administered before MI ablation. The MI ablation was conducted during CS pacing for real‐time conduction assessment. However, in contrast to Cohort A, the initial MI ablation series consisted of 20 PFA applications (instead of nine), with 16 applications in the “flower” configuration and the final four in the “basket” configuration. During ablation in the flower configuration, the pentaspline catheter was slightly rotated (approximately 10°–25°) after every four applications to enhance lesion overlap. As in Cohort A, distal CS or LA appendage pacing was performed for 20 min to verify the bidirectional block, and the procedure was concluded if the bidirectional MI block persisted. If reconduction appeared during the observation period, a second identical series of 20 applications was delivered, followed by another 20‐min observation period. If a persistent block was still absent after the second series of 20 applications, an optional third and final ablation series could be applied. The number of pulses and the configuration of the catheter during the third series were also left at the electrophysiologist's discretion; however, as with Cohort A, the same safety limit for the total number of MI PFA applications (i.e., 50) and total observational time (i.e., 40 min) was followed.

In patients with a history of typical atrial flutter, CTI ablation was performed as the last step, that is, after completion of all LA ablations. Ablation was done in either the flower or the flower and basket configuration. No prophylactic nitrates were administered before CTI ablation. The goal was a bidirectional CTI block.

### 2.6 Post‐Procedural Care

Post‐procedurally, patients were monitored in the cardiac intensive care unit for a minimum of 3 h and discharged the next day. A standardized intravenous hydration protocol consisting of 1000 mL of normal saline was administered. Low molecular weight heparin at a dose of 1 mg/kg was administered when the activated clotting time dropped below 150 s, and oral anticoagulation was reinitiated the morning of the following day. Renal function parameters, including blood urea nitrogen and serum creatinine, were assessed on the first post‐procedural day.

### 2.7 Study Endpoints

The primary efficacy endpoint was defined as achievement of a durable first‐pass bidirectional MI conduction block following the 20‐min observation period (*first‐pass durable MI block*). The secondary endpoint was defined as durable mitral isthmus conduction block following completion of the full protocol‐defined ablation strategy and verified after a 20‐min observation period after the final ablation series (*final durable MI block*).

Safety endpoints were defined as the occurrence of ST‐segment changes on electrocardiography during mitral isthmus ablation and the development of AKI on post‐procedural day one.

### 2.8 Follow‐Up

All patients underwent follow‐up to assess AF or atrial tachycardia/atrial flutter recurrence at 3 months. Follow‐up information was obtained through review of the medical record, communication with treating cardiologists, or direct telephone contact.

### 2.9 Statistical Considerations

Continuous data were reported as means and standard deviations (SD), medians and interquartile ranges (IQR) for non‐parametric data and counts and percentages for categorical data. The normality of data distribution was assessed using the Shapiro–Wilk test. Categorical data, including efficacy endpoints, were compared using Fisher's exact test. Quantitative data were compared using the Student's *t*‐test when the data had a normal distribution; otherwise, the Mann–Whitney *U* test was used.

All analyses were performed using R Statistical Software (v4.3.3; The R Foundation for Statistical Computing, 2024).

The study was not randomized. The analysis of Cohort A was descriptive, with no formal hypothesis or power calculation. Regarding Cohort B, the sample size was calculated after the enrollment to Cohort A were concluded and the results were available. Since only 33% of patients had a first‐time durable MI block after the 20‐min observation period, whereas 90% of patients achieved a durable block after the final ablation series, we expected to increase the rate of a first‐pass durable MI block to 65% of patients by increasing the number of PFA applications per series to 20. This assumption was based not only on the results of Cohort A, but also on emerging evidence suggesting that deeper lesions can be achieved by increasing the number of PFA applications [12]. Accordingly, 40 patients were enrolled in Cohort B to detect a statistically significant difference relative to first‐time MI block compared to Cohort A.

## 3 Results

### 3.1 Patients and Procedures

Between September 2024 and January 2025, a total of 30 patients were prospectively enrolled into Cohort A. The mean age was 68.3 ± 6.3 years; 11 patients (36.7%) were female.

Between January and June 2025, a total of 40 patients were prospectively enrolled in Cohort B. The mean age was 67.9 ± 7.6 years; 15 (37.5%) patients were female. The baseline characteristics of the two cohorts were similar (Table 1).

**Table 1:** Baseline characteristics.

	Cohort A	Cohort B	*p* value
Number of patients	30	40	
Female	11 (36.7%)	15 (37.5%)	1.00
Age, y	68.3 ± 6.3	67.9 ± (7.6)	0.80
BMI, kg/m^2^	28.9 (IQR 26.1–32.6)	30.1 (IQR 27.1–32.9)	0.50
Chronic heart failure	10 (33.3%)	11 (27.5%)	0.61
Hypertension	23 (76.7%)	30 (75%)	1.00
Diabetes	12 (40%)	13 (32.5%)	0.62
Atherosclerotic vascular disease	7 (23.3%)	10 (25%)	1.00
History of stroke/TIA	1 (3.3%)	5 (12.5%)	0.23
CHA_2_DS_2_‐VASc score	3.0 ± 1.3	2.9 ± 1.3	0.70
Time from initial AF diagnosis, months	17.5 (IQR 7.3–41.5)	12.5 (IQR 7.5–35.3)	0.71
Beta‐blockers	24 (80%)	31 (77.5%)	1.00
Antiarrhythmic drugs	15 (50%)	25 (62.5%)	0.34
Propafenone	4 (13.3%)	12 (30%)	0.15
Amiodarone	11 (36.7%)	13 (32.5%)	0.80
LVEF, %	55 (IQR 55–60)	55 (IQR 50–60)	0.39
LA diameter, mm	45.8 ± 5.0	46.7 ± 4.7	0.41
LAVi, mL/m^2^	48.9 ± 12.7	48.8 ± 10.1	0.97

*Note:* Baseline characteristics of patients in Cohorts A and B.

Abbreviations: BMI = body mass index, IQR = interquartile range, LA = left atrium, LAVi = left atrial volume indexed to the body surface area, LVEF = left ventricular ejection fraction, TIA = transient ischemic attack.

In Cohort A, the median total number of PFA applications was 78.0 (IQR 72.0–87.0), of which 35.0 (IQR 32.0–38.0) were administered to the pulmonary veins, 23.0 (IQR 20.0–28.0) to the posterior wall of the left atrium, and 18.0 (IQR 9.0–34.0) to the MI. Two (6.7%) patients developed typical right atrial flutter and underwent cavotricuspid isthmus ablation during the same procedure. Three (10.0%) patients developed peri‐mitral re‐entry atrial tachycardia during the procedure (Table 2).

**Table 2:** Procedural characteristics.

	Cohort A	Cohort B	*p* value
Length of the procedure, min	82.6 ± 13.6	88.9 ± 14.2	0.07
General anesthesia	6 (20.0%)	28 (70.0%)	0.001
LA dwelling time, min	65.1 ± 15.2	60.7 ± 9.5	0.15
PFA applications			
Total	78.0 (IQR 72.0–87.0)	80.0 (IQR 75.0–94.0)	0.26
Pulmonary veins	35.0 (IQR 32.0–38.0)	36.0 (IQR 32.0–38.0)	0.74
Posterior wall	23.0 (IQR 20.0–28.0)	22.0 (IQR 18.0–24.5)	0.07
Mitral isthmus	18.0 (IQR 9.0–34.0)	20.0 (IQR 20.0–40.0)	0.004
CTI ablation	2 (6.7%)	5 (12.5%)	0.69
Peri‐mitral re‐entry during procedure	3 (10.0%)	3 (7.5%)	1.00
Fluoroscopy time, min	5.6 (IQR 4.3–6.4)	8.4 (IQR 6.5–12.1)	< 0.001
Fluoroscopy dose, mGy. cm^2^	2452 (IQR 1938–2984)	4039 (IQR 3328–4729)	< 0.001

*Note:* Procedural characteristics.

Abbreviations: CTI = cavotricuspid isthmus, LA = left atrium, PFA = pulsed‐field ablation.

In Cohort B, the median of total PFA applications was 80.0 (IQR 75.0–94.0), of which 36.0 (IQR 32.0–38.0) were administered to the pulmonary veins, 22.0 (IQR 18.0–24.5) to the posterior wall of the left atrium, and 20.0 (IQR 20.0–40.0) to the MI. Two patients (5.0%) developed typical right atrial flutter, and a total of 5 (12.5%) patients underwent cavotricuspid isthmus ablation during the same procedure. Three patients (7.5%) developed peri‐mitral re‐entry atrial tachycardia during the procedure (Table 2).

### 3.2 Efficacy Endpoint Assessment

#### 3.2.1 Cohort A

Immediately after the last PFA application of the first series, a MI bidirectional block was present in 26 patients (86.7%). However, due to reconductions occurring during the observation period, a first‐pass durable MI block, confirmed after the 20‐min observation period, was only present in 10 (33.3%) patients. A final durable MI block, that is, a durable bidirectional MI block after the completion of the full ablation protocol, was present in 27 (90%) patients.

**Figure 3:**
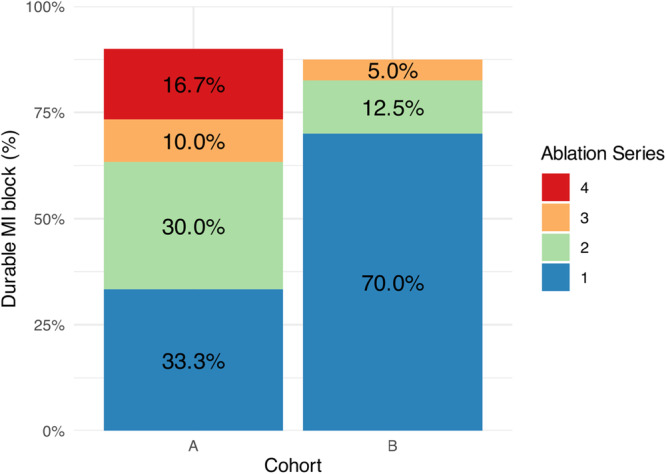
Success rate of durable mitral isthmus conduction block after each ablation series in Cohorts A and B.

In detail, a durable bidirectional MI block was observed in an additional nine (30%) patients after the second series, three (10%) patients after the third series, and five (16,7%) patients after the fourth series (Figure 3). A durable MI block was not achieved in three patients (10%).

**Figure 4:**
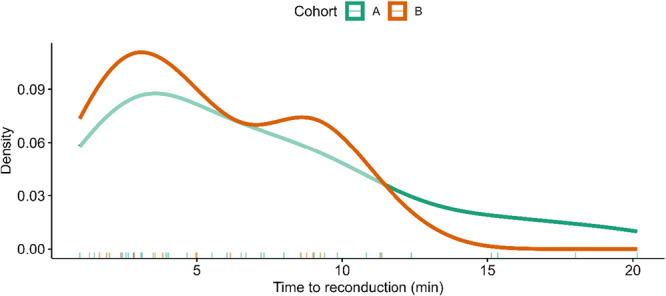
Kernel density plots illustrating the temporal distribution of reconduction events for each cohort (*y*‐axis: Kernel Density Estimate, *x*‐axis: individual event time points). In Cohort A, the maximum of reconductions through MI occurred approximately 3 min after MI achievement, decreasing continuously over time, with a few reconductions documented much later (15–20 min). In contrast, Cohort B exhibited two peaks of MI block reconductions: one occurring within 5 min and the other within 10 min. Notably, no reconduction events were observed later than 15 min after the MI block was achieved in cohort B.

In patients with reconduction, the median time to reconduction was 6.0 (IQR 2.8–9.4) minutes (Figure 4). Reconduction occurred within the first 10 min of the observation period in 78.8% of cases.

#### 3.2.2 Cohort B

Immediately after the last PFA application of the first series of applications, a MI bidirectional block was present in 38 patients (95%). However, similar to Cohort A, reconduction during the 20‐min observation period reduced the confirmed first‐pass MI block rate to 28 patients (70%). The rate of the first‐pass MI block in Cohort B was significantly higher than in Cohort A (*p* = 0.003). After completing the full ablation protocol, a final durable MI block was present in 35 patients (87.5%), a rate similar to that of Cohort A (*p* = 1.00).

In detail, an MI block was achieved in five (12.5%) more patients after the second series of PFA applications. Of the seven patients with reconduction after the second series, only three received the optional third ablation series, and of these, two achieved a durable conduction block. As a result, the final cumulative rate of durable bidirectional MI block after the second and third series was 82.5% and 87.5%, respectively (Figure 3).

In patients with early reconduction, the median time to reconduction was 5.0 min (IQR 2.6–8.7). In 93.3% of patients, reconduction appeared within the first 10 min (Figure 4). Time to reconduction was similar between the two cohorts (*p* = 0.39).

### 3.3 Safety

There were no major complications in Cohort A or B; there were no deaths, no pericardial effusions, and no strokes or transient ischemic attacks. Predefined safety endpoints occurred in two (2.9%) patients. Both were cases of ST‐segment depression during ablation of the MI and lasted 3 and 11 min, respectively. In both patients, the ECG changes resolved spontaneously without the need for nitrates and did not result in hemodynamic consequences (Table 3).

**Table 3:** Safety endpoints and complications.

	Cohort A	Cohort B	Total
Predefined safety endpoint	0	2 (5%)	2 (2.9%)
ST‐segment changes during MI ablation	0	2 (5%)	2 (2.9%)
AKI on postprocedural Day 1	0	0	0
Major complications (death, pericardial effusion, stroke/TIA)	0	0	0
Minor complications	0	0	0
Vascular access complications	1 (3.3%)	0	1 (1.4%)
Other (see text)	2 (6.7%)	0	2 (2.9%)
All	3 (10%)	2 (5%)	5 (7.1%)

*Note:* Safety endpoints and complications.

Abbreviations: AKI = acute kidney injury, MI = mitral isthmus, TIA = transient ischemic attack.

There were no AKIs on post‐procedural Day 1. Overall, we did not observe a rise in serum creatinine on post‐procedural Day 1 compared with preprocedural values, with a mean change in serum creatinine of −6.0 ± 9.5 μmol/L in Cohort A and −4.8 ± 10.3 μmol/L in Cohort B. In Cohort A, the median baseline s‐creatinine was 88.5 (IQR 72.0–97.5) μmol/L and declined to a median of 82.0 (IQR 72.0–95.0) μmol/L on post‐procedural Day 1. In Cohort B, the median baseline s‐creatinine was 79.5 (IQR 67.8–105.0) μmol/L and declined to a median of 75.5 (IQR 68.5–89.0) μmol/L on post‐procedural Day 1.

There was one vascular access complication—a pseudoaneurysm in the groin requiring a thrombin injection. There was also one case of symptomatic bradycardia after the procedure, requiring temporary treatment with isoproterenol and resolving within hours without consequences.

### 3.4 Follow‐Up

Follow‐up data were available for 69 patients (98.6%). AF recurrence at 3 months occurred in 5 patients (17.2%) in Cohort A, and 7 patients (17.5%) In Cohort B. Regular atrial tachycardia/atrial flutter occurred in 2 patients (6.9%) and 4 patients (10.0%) in Cohort A and Cohort B, respectively.

A total of four patients, with two from each cohort, underwent a redo procedure due to the development of atypical atrial flutter. In all cases, perimitral flutter was observed, with reconduction occurring through the MI. This was successfully ablated in each instance. In three of the patients, the initial procedure concluded with a durable MI block, confirmed after a 20‐min waiting period. However, in one case, an MI block was not achieved during the initial procedure.

## 4 Discussion

In our study, the ablation setting of 20 PFA applications per series was associated with a significantly higher rate of first‐pass MI block that persisted after a 20‐min waiting period, compared to the setting of only nine applications per series (70% vs. 33%, *p* = 0.003). Interestingly, the rate of final MI block was similar at both ablation settings (90% and 87.5%, *p* = 1.00).

Notably, a bidirectional block was almost always present immediately after ablations had been delivered, with reconductions occurring during the 20‐min observation period. Additionally, it is important to note that reconduction usually occurred during the first 10 min of the observation period, with a median duration of 6.0 and 5.0 min in Cohort A and B, respectively. No major complications were observed in either Cohort.

The Mitral isthmus represents a particularly challenging target for catheter ablation due to increased wall thickness and proximity to the circumflex coronary artery and the great cardiac vein, which functionally increases the thickness of the LA wall due to its epicardial connections [13]. Using RF energy, ablation of the mitral isthmus line frequently requires not only extensive endocardial ablation, but also epicardial ablation from within the coronary sinus. When using RF energy, the success rate of endocardial ablation alone has been reported at ~32% [5, 6]. When combined with epicardial RF ablation from within the CS, an MI block can be achieved in 56%–92% of patients [5, 14, 15]. Ethanol infusion into the vein of Marshall further increases the success rate up to 78%–96.4% [1, 6, 14]. However, all these strategies substantially prolong the procedure and increase risks.

Our ablation target was the lateral MI. To optimize lesion effectiveness, we positioned the mitral lesion more anterior relative to the conventional line because myocardial thickness is less in this area, and the occurrence of a muscular venous sleeve is minimal. Furthermore, due to branching, the great cardiac vein is narrower in the anterosuperior aspect of the mitral anulus, effectively reducing the required depth of the ablation lesion [16]. A myocardial depth of 3.6 ± 0.8 mm has been described for the LA aspect ablated using our approach [16, 17].

In contrast to a large number of reports describing MI ablation using RF energy, few reports have been published on PFA of the lateral MI. Therefore, many unresolved issues remain in MI ablations using PFA, such as determining the optimal ablation site, the number of PFA lesions required, the need for observation period, and the duration of observation. Initial reports describing the use of PFA beyond PVI were enthusiastic, with some reporting rates of persistent MI block of 100% [10]. However, in this report, the presence of an acute block immediately after the last PFA application was considered a success. Later studies, which included a post‐ablation observation period, and reports on redo procedures after PFA, revealed reconductions, especially outside the PVs.

Reddy et al. reported a series of 65 patients who underwent ablation using a lattice‐tip catheter. Twenty‐two patients also underwent a posterior MI line ablation, achieving 100% acute success (note that this was assessed immediately after the procedure without an observation period). LA remapping was done 3 months later in 11 patients, and the bidirectional MI block was present in 10 (91%) [18]. Similarly, Vetta et al. reported a first‐pass MI block after endocardial PFA in 38 of 45 (84.4%) AF patients, also using a lattice‐tip catheter. At the end of the full procedure, a MI block was present in 100% of patients; however, the last seven patients required further PFA within the coronary sinus, and RFA was used in 17.8% of patients [19]. No late remapping was done, so no information on lesion durability is available. In contrast to our study, both these reports used a different catheter, one that enables delivery of both RF and pulsed‐field energy.

Regarding the pentaspline catheter, Zhang et al. reported a series of 17 patients with 100% success after MI ablation with a mean of 10.5 ± 3.9 PFA applications on the posterolateral MI. However, the bidirectional block was assessed immediately after the last PFA application, without any observation period [10]. This remarkably high immediate success rate aligns with our findings.

Davong et al. reported a series of 45 patients who underwent PFA of the MI posterior line using a pentaspline catheter as a standard part of the procedure. Acute complete MI block was achieved in all patients; however, after a 20‐min observation period, the MI block only persisted in 86.7% of patients. Notably, the ablation protocol did not specify a predefined number of PFA applications; however, the mean number of applications delivered to the MI was relatively high (31.2 ± 17.5). Although the exact times of reconduction were not reported, the observed recurrence of conduction emphasized the need for a standard observation period after MI ablations [20]. In agreement with the study by Davong et al., we achieved immediate bidirectional MI block in the vast majority of patients, but a significant number of reconductions occurred during the observation period, which was dependent on the number of PFA applications.

An observation period of 20 min is standard after the cavotricuspid or mitral isthmus ablation using RF energy. The need for an observation period after PFA and its optimal duration remain unsettled. Nor is the precise mechanism of reconduction known, since many blocks after PFA seem to be also only transient, which is similar to that seen in RF ablations. Further research is needed to determine the optimum observation period after PFA. Although the optimum duration of the observation period after PFA is not known. It is noteworthy that the rate of reconduction in our study declined over time. In Cohort B, reconduction after 10 min only occurred in 6.7% of cases, and none occurred after 15 min. Therefore, it appears that, similar to RF ablations, the highest incidence of reconduction occurs immediately after the last application of energy and decreases over time (Figure 4).

In the context of PFA, early reconduction is likely attributable to reversible electroporation, in contrast to the resolution of local edema in RFA. This phenomenon occurs when the local electric field fails to reach the threshold for irreversible cellular injury, permitting cardiomyocyte recovery. Notably, the loss of voltage cannot predict the durability of lesions since voltage abatement was observed even in cardiac tissue that had recovered after PFA, with no difference in voltage reduction compared to durable lesions resulting in fibrosis [21]. In an animal study, the histology of tissues showing transient electrogram amplitude reduction after PFA found minimal to no fibrosis. Based on this concept, delivery of a subtherapeutic dose of pulsed‐field energy to induce a transient conduction block has even been proposed as a mapping tool [22].

As recently shown in another animal study, the number of PFA applications plays a very significant role in lesion formation. Di Biase et al. studied the effect of contact force and lesion number on lesion formation (lesion depth and width) in an animal model using an OMNYPULSE PFA catheter. Lesion depth increased significantly with both higher contact force and a greater number of PFA applications. At minimum contact force, depth rose from 2.12 ± 0.71 mm with three applications to 3.92 ± 0.91 mm with 12 applications. At maximum contact force, the increase was even more pronounced, from 3.34 ± 1.43 mm with three applications to 5.32 ± 0.77 mm with 12 applications [12]. This observation is fully in line with our results. A greater number of applications in Cohort B was linked to a higher likelihood of achieving a successful first‐pass block. While there were some minor differences in ablation techniques between the two cohorts—such as the use of basket configuration and varying levels of operator experience—the number of sequential applications seems to play a significant role. The number of patients with a final persistent block was similar in both Cohorts, corresponding to the similar final number of PFA applications.

The long‐term durability of lateral MI ablation was assessed in a recent work by La Fazia et al. In this study, complex left atrial ablation was performed with a pentaspline catheter in 236 patients, including lateral MI ablation. Acute MI reconduction was observed in 14.8% of cases after a 20‐min waiting period and adenosine challenge. At 3‐month remapping, a durable MI block was maintained in only 5.5% of patients. While these findings raise questions regarding the long‐term utility of lateral MI ablation, several methodological considerations should be noted. First and most importantly, MI block during the index procedure was assessed only by the elimination of local electrograms, which might significantly overestimate the acute success rate. Second, in cases of reconduction after the observation period, only four additional applications were delivered with no subsequent reassessment of the MI block. Third, remapping procedures only involved voltage mapping rather than activation mapping, which is considered the gold standard for confirming line block [23].

### 4.1 Safety Concerns

One of the concerns regarding PFA in proximity to a major epicardial artery is the risk of coronary artery spasm, which has a reported incidence of 4.4% in mitral isthmus ablations [20]. The spasms caused by PFA are, in the majority of cases, transient, having no hemodynamic consequences. Still, rare severe spasms resulting in hemodynamic deterioration or induction of ventricular arrhythmia have been described [10, 24]. In our patients, despite the absence of prophylactic nitrate before PFA, only two (2.9%) experienced transient ST‐segment changes, all without clinical consequences.

Another safety concern during PFA is the risk of hemolysis and subsequent AKI. The risk increases with the number of PFA applications, particularly above 70 [25]. With extensive ablation of the mitral isthmus, cumulative PFA applications can easily surpass this threshold. In our study, the median number of PFA applications was 78.0 (IQR 72.0–87.0) in Cohort A and 80.0 (IQR 75.0–94.0) in Cohort B. Despite these elevated counts, no cases of AKI were observed on the day following the procedure. Careful patient selection, periprocedural parenteral hydration, and limiting the number of PFA applications in patients with pre‐procedural kidney disease can mitigate these risks.

### 4.2 Study Limitations

Our study is subject to several limitations. First, the sample size was modest, which can limit the power to detect infrequent complications as well as subtle differences between the two PFA strategies employed. Second, the study was not randomized. Third, electroanatomical mapping was not performed during the index procedure. Finally, although our study involved a 20‐min observation period, no routine late reassessment of the MI block durability was included.

## 5 Conclusions

MI ablation using a pentaspline PFA catheter is both feasible and safe and can be achieved in up to 90% of patients with a first‐pass success rate of 70% when using a strategy of 20 applications per series. The vast majority of reconductions occur within the first few minutes following ablation; an observation period of 20 min after an ablation series appears well‐justified and clinically appropriate.

## Funding

The authors received no specific funding for this work.

## Ethics Statement

The protocols for each cohort were reviewed and approved by the local institutional ethics committees.

## Consent

All participants provided written informed consent before enrollment.

## Conflicts of Interest

The authors declare no conflicts of interest.

## Data Availability

The data underlying this article will be shared at a reasonable request to the corresponding author.
